# Prevalence and Factors Associated with HIV Testing Among Women of Reproductive Age in Liberia: A Cross-Sectional Study from the 2019/20 Demographic and Health Survey

**DOI:** 10.3390/ijerph22081234

**Published:** 2025-08-07

**Authors:** Mapei Mary Anna Kolane, Lumbani Tshotetsi

**Affiliations:** 1 School of Health Systems and Public Health, Faculty of Health Sciences, University of Pretoria, Pretoria 0028, South Africa; u22541994@tuks.co.za; 2 Clinical Associate Program, Department of Family Medicine, Faculty of Health Sciences, University of Pretoria, Pretoria 0028, South Africa

**Keywords:** HIV testing, factors, reproductive age, women, Liberia, prevalence

## Abstract

*Objective:* This study explored HIV testing prevalence and its associated factors among reproductive-aged women in Liberia. *Study Design:* A secondary and descriptive cross-sectional study was performed among Liberian women aged 15–49 years using the 2019 Liberia Demographic and Health Survey (LDHS) data set. *Methods:* Descriptive statistics were used to describe the characteristics of these women. Bivariate and multivariable logistic regression models were applied to determine factors associated with HIV testing. All analyses were adjusted for unequal probabilities of selection and non-response by use of survey weights. *Results:* Among the 8065 participants in this survey, 490 women had never had sex and were excluded, leading to the final sample size being 7575 women. The prevalence of HIV testing among Liberian women aged 15 to 49 years in 2020 was 57.17% (95% CI: 56.2 to 60.4). HIV testing among these women is associated with pregnancy history (aOR 6.40, 95% CI:4.99 to 8.22, *p* < 0.001), STI history (aOR 1.21, 95% CI:1.02 to 3.19, *p* < 0.001), knowledge of vertical transmission (aOR 1.65, 95% CI:1.23 to 2.21, *p* = 0.001), and highest educational level (primary (aOR 1.39, 95% CI:1.16 to 1.68, *p* < 0.001), secondary (aOR 2.10, 95% CI:1.73 to 2.53, *p* < 0.001), and higher education (aOR 6.80, 95% CI:3.75 to 12.32, *p* < 0.001)). *Conclusions and Contribution:* HIV testing prevalence of 57.17% demonstrates an unmet need for HIV testing among Liberian women aged 15 to 49 years and, thus, it is recommended that HIV testing and counseling services should mostly target these women in rural areas, with limited health services and less educated women.

## 1. Background

Every country’s mandate is to provide universal health coverage for all her people. Globally, approximately 38 million people were living with HIV in 2019, with more than 75% living in sub-Saharan Africa [1]. Approximately 85% of the people living with HIV globally knew their HIV status in 2021, while the remaining 15% did not know they had HIV and still needed access to HIV testing services [1,2]. In 2019, approximately 690,000 people died of AIDS-related causes and 1.7 million people were newly infected with HIV [1]. In sub-Saharan Africa, women and girls accounted for 63% of all new HIV/AIDS infections in 2021 [2]. In 2018, it was reported that 65% of the people living with HIV (PLHIV) in Liberia knew their HIV status [2]. Improvement in strategies to get more people tested for HIV in Liberia will increase antiretroviral treatment (ART) initiation and viral load suppression, which will result in an optimal route in meeting the UNAIDS 95-95-95 targets [3].

Furthermore, HIV testing is the main gateway for HIV prevention, care and treatment [4]. Globally, HIV testing is crucial in building effective strategies toward reducing HIV/AIDS prevalence [5,6,7,8,9]. Previous studies have demonstrated that HIV testing challenges can be addressed through managing certain potential risk factors associated with HIV testing [10,11,12,13,14]. Most importantly, there has always been an unmet need for HIV testing among Liberian women [1].

Among others, risk factors for HIV testing include knowledge about vertical transmission, pregnancy history, sexually transmitted infection (STI) history, and higher educational levels for women and their partners [4]. Most studies focusing on HIV testing have been performed on certain subgroups of women who form part of the high-risk populations such as adolescent girls and young women (AGYW) [15,16,17,18,19]. However, this may exclude certain attributes of all women of reproductive age and, thus, less informed conclusions may be extracted. This study determined the prevalence and associated factors of HIV testing among Liberian reproductive-aged women.

HIV testing has not been documented among Liberian women. A previous study has documented HIV testing prevalence and its associated risk factors within a single health center in Liberia [1]. This might have omitted important characteristics which could have otherwise redirected policymakers towards implementing efficient strategies to reduce HIV/AIDS and thus improving SDG 3, universal health coverage in Liberia. Furthermore, slow regression in the prevention of mother-to-child transmission (MTCT) of HIV has been observed in Liberia from about 28% in 2011 to 15% in 2018 [2].

Most importantly, Liberia has taken the initiative to respond effectively to the HIV pandemic. Liberia’s Ministry of Health, in collaboration with the National AIDS Commission of Liberia, has developed a fast-track plan for 2019/2020 that seeks to triple the country’s test and treat statistics by treating people who test positive for HIV immediately after diagnosis [3]. This plan includes targeting all population groups most at risk of HIV infection with the inclusion of women and the three counties with the highest unmet need for HIV testing, treatment and care services [2].

Overall, this calls for a review of strategies of HIV testing among women. Thus, this study aims to address these gaps and determine the prevalence and associated factors of HIV testing among the entire population of Liberian reproductive-aged women.

## 2. Methodology

### 2.1. Study Design

This study is a secondary data analysis of the 2019–2020 Liberia Demographic and Health Survey (LDHS), a nationally representative, population-based analytical cross-sectional survey, conducted to assess the prevalence and factors associated with HIV testing among reproductive-aged women in Liberia (15–49 years).

### 2.2. Study Setting

Data were collected from all 15 counties in Liberia, which were grouped into five geographical regions, each comprising three counties. Each county is subdivided into districts, which are further divided into clans. According to the 2008 National Population and Housing Census (NPHC), each clan was segmented into enumeration areas (EAs). The LDHS sample design ensured that results were representative at the national level, for urban and rural areas, and across the five regions. Additionally, the survey provided separate, representative estimates for most key indicators across the 15 counties [8].

### 2.3. Study Population

This study included all reproductive-aged Liberian women (15–49 years) who were either usual residents of the selected households or were present the night before the survey interview. A household was defined as a group of individuals who usually live together and share meals, including both usual residents and overnight visitors. In these households, all women aged 18–49, as well as emancipated minors or those with parental or guardian consent, were eligible for HIV testing. For this analysis, women who had never heard of HIV/AIDS (*n* = 5%) and those who reported never having had sexual intercourse (*n* = 7%) were excluded [20].

### 2.4. Sampling Method

The 2019–2020 LDHS employed a stratified two-stage cluster sampling design. In the first stage, 325 clusters were selected from EAs using probability proportional to size within each sampling stratum. In the second stage, an average of 129 households were listed in each cluster, from which a fixed number of 30 households were systematically selected with equal probability. A total of 9068 households were successfully interviewed. Within these households, 8364 women aged 15–49 years were identified for individual interviews, and 8065 women completed the interview [4].

### 2.5. Measurements and Variables

This study employed data collection using the Women’s Questionnaire, administered to reproductive-aged women (15–49 years). It further involved secondary analysis on the dependent variable HIV testing (no, yes) and the predictor variables: age of a woman (15–19, 20–24, 25–29, 30–34, 35–39, 40–44, 45–49), educational level (no education, primary, secondary, higher education), place of residence (urban, rural), marital status (married, not in union, living with a man, widowed, divorced/separated), media (TV, radio, newspapers/magazines), exposure (not at all, less than once a week, more than once a week), partner’s highest educational level (no education, primary, secondary, higher education), employment status (not employed, employed), pregnancy history (no, yes), number of sexual partners (1 partner, 2 partners, 3 or more partners), transactional sex (no, yes), STI history (no, yes), HIV knowledge (no, yes), knowledge of PMTCT (no, yes) and HIV discriminatory behavior (no, yes) [8].

### 2.6. Statistical Analyses

All analyses were performed using Stata MP 14.0 software. All analyses were weighted to adjust for unequal probabilities of selection and non-response. Descriptive statistics were used to summarize the characteristics of participants. Bivariate analysis was employed to check for association between the HIV testing outcome and each independent variable using the Chi-square test. Variables with *p* < 0.05 were shortlisted and entered onto the multivariable logistic regression model. In the multivariable logistic regression model, manual selection was used to select variables into the multivariable analysis. Crude and adjusted odds ratios, together with their corresponding 95% confidence intervals (CI), were tabulated and a 5% level of significance was applied.

### 2.7. Ethical Considerations

The research protocol was submitted to the University of Pretoria School of Health Systems and Public Health (SHSPH) Academic Advisory Committee (AAC) for approval. It was then approved by the University of Pretoria’s Faculty of Health Sciences Research Ethics committee (136/2023). Since this is secondary data analysis, all the Demographic and Health Survey (DHS) ethical considerations were adopted. Finally, permission to use DHS data was obtained from the DHS online database and an agreement of the terms and conditions of using the data set was signed.

## 3. Results

### 3.1. Study Participants

Among the 8065 women aged 15 to 49 years who were interviewed in this survey, 490 women who had never had sex were excluded in this analysis, leading to the final sample size of 7575 women.

### 3.2. Characteristics of Participants

Among the 7575 included reproductive-aged women, most of them were young, aged 20 to 24 years (19.6%) and 25 to 29 years (18.3%). About 39.8% of these women had at most a secondary education qualification and mostly resided in urban areas (61.7%). About 34.3% of these women were living with a man. More than three quarters of them were never exposed to media (78.2%). About two thirds of them were not employed (67.1%). Almost half of them had partners with at most a secondary qualification (47.0%). The majority of these women had been pregnant at least once in their lifetime (83.2%). Most had one partner (92.7%) and had never been involved in transactional sex (91.7%). Almost all had never experienced gender-based violence (98.0%), while most women possessed discriminatory behavior towards HIV-positive people (98.8%), as shown in Table 1.

### 3.3. Factors Associated with HIV Testing

Among 7575 women who were included in this study, 4331 women had tested for HIV at least once. The HIV testing prevalence was 57.17% (95% CI: 56.2 to 60.4) among women aged 15 to 49 years.

The likelihood of HIV testing increased with age, peaking among women aged 35–39. After adjusting for confounding variables, women aged 20–39 had significantly higher odds of testing compared to those aged 15–19, with aORs ranging from 1.58 (95% CI: 1.05–2.38, *p* = 0.028) in the 20–24 group to 1.95 (95% CI: 1.20–3.17, *p* = 0.007) in the 35–39 group. The association weakened for older groups and was not statistically significant for ages 40–49, as shown in Table 2.

Educational attainment was significantly associated with higher odds of HIV testing. Compared to women with no education, the odds remained significantly higher for those with primary (aOR 1.39, 95% CI: 1.16–1.68, *p* < 0.001), secondary (aOR 2.10, 95% CI: 1.73–2.53, *p* < 0.001), and higher education (aOR 6.80, 95% CI: 3.75–12.32, *p* < 0.001).

Women residing in rural areas had lower odds of HIV testing than their urban counterparts, with the adjusted analysis confirming statistical significance (aOR 0.82, 95% CI: 0.67–0.99, *p* = 0.039).

Marital status also played a significant role. Compared to married women, those living with a man (aOR 1.35, 95% CI: 1.03–1.51, *p* < 0.001) and those who were divorced or separated (aOR 1.37, 95% CI: 1.05–1.79, *p* = 0.019) had higher odds of having tested for HIV. No significant differences were observed for women not in union or widowed.

Regarding media exposure, no significant associations were found in the adjusted analysis. While women with more frequent media exposure had higher unadjusted odds of HIV testing, the adjusted odds were not statistically significant (aOR 0.81, *p* = 0.606 for less than once/week; aOR 1.66, *p* = 0.096 for more than once/week).

Partner’s education was positively associated with HIV testing among women. Those whose partners had secondary (aOR 1.41, 95% CI: 1.13–1.75, *p* = 0.002) or higher education (aOR 1.82, 95% CI: 1.13–2.93, *p* = 0.013) were significantly more likely to test for HIV (Table 2).

### 3.4. Pregnancy History

Among 4331 women who tested for HIV, 93.35% had been pregnant at least once in their lifetime. Compared to women who were never pregnant, women with a history of pregnancy had substantially higher odds of testing, and this association remained highly significant in adjusted analysis (aOR 6.40, 95% CI: 4.99–8.22, *p* < 0.001) (Table 2).

### 3.5. STI History

Among the 4327 women who tested for HIV, about 31.94% had STIs. Compared to women who never had STIs, those with STIs had significantly higher odds of HIV testing in adjusted analysis (aOR 1.21, 95% CI: 1.02–1.50, *p* = 0.030) (Table 2).

### 3.6. Knowledge of MTCT

Knowledge about the availability of medication to prevent MTCT of HIV was significantly associated with testing. Women who knew about MTCT drugs had higher odds of testing even after adjustment (aOR 1.65, 95% CI: 1.23–2.21, *p* = 0.001), as shown in Table 2.

## 4. Discussion

This study aimed to determine the prevalence and factors associated with HIV testing among women aged 15 to 49 years in Liberia using 2019/2020 DHS data.

The prevalence of HIV testing among women aged 15 to 49 years in Liberia was found to be 57.17% (95% CI: 56.2 to 60.4). This prevalence was higher than the one reported in sub-Saharan Africa [21] but lower than many studies conducted in Africa [6,19].

Regional variations in access to HIV testing facilities and knowledge related to HIV/AIDS likely contribute to the observed disparities in HIV testing across Liberia [20,21,22,23]. According to the 2019–2020 LDHS, the highest testing coverage among women aged 15–49 was in the South-Central region (78.3%) and lowest in Southeastern B (42.4%). These differences reflect unequal access to healthcare services and education, with urban areas like Montserrado benefiting from better infrastructure [20]. Additionally, Liberia’s prolonged civil unrest has left a weakened health system and critical shortages in the health workforce, further impacting service delivery [2].

This study found that women who live in rural areas have lower HIV testing prevalence compared to those who live in urban areas. This might be because women who live in urban areas can easily access primary healthcare services and have better exposure to accurate information and educational programs about HIV/AIDS [24]. In a rural setting, especially in very small villages, lack of privacy and confidentiality of healthcare personnel may also reduce the rate of HIV testing. Apart from that, cultural and religious beliefs in rural areas discourage discussions about sexual health, which may impede HIV testing processes [11,24]. This was contrary to a finding by Deynu et al. who reported that most women from rural areas are more likely to test for HIV than those from urban areas [25].

Women in urban areas are more educated compared to rural areas. We observed that there is a strong association between HIV testing among these women and their highest educational level. Women who had a higher education qualification were more likely to test for HIV than the less educated group in this study. This could have been attributed to better comprehension of the importance of HIV testing and its risk factors, access to information, financial ability to seek healthcare services and reduced stigma [26]. This, in return, may decrease women’s fear of using HIV testing services. Bhattarai et al. also reported that having primary, secondary, or higher education was associated with increased odds of HIV testing compared to those with no education [23].

Access to HIV testing is also influenced by contact with the health system, particularly for reproductive and maternal care. Women who had been pregnant at least once in their lifetime had higher odds of testing for HIV. This may be motivated by routine HIV testing during antenatal care to prevent vertical transmission of HIV. Knowledge about prevention of mother-to-child transmission (PMTCT) also positively influenced HIV testing, as it empowers women to protect their children and can also encourage partner testing. Pachena & Musekiwa found that women with more knowledge about MTCT had higher odds of being tested for HIV [26]. Furthermore, Sonny & Musekiwa reported that knowledge of MTCT was associated with ever testing for HIV in Lesotho [12]. A study in Zimbabwe by Pachena & Musekiwa also found that adolescent girls and young women (AGYW) who had been pregnant in the past 24 months had higher odds of HIV testing [27].

Women who had a history of STIs were more likely to have tested for HIV than those without such history. This may be due to increased awareness of HIV risk among STI patients and the frequent contact with health services that facilitates HIV testing opportunities. However, despite this observed association, our data revealed a high prevalence of STIs among the study population, even though the majority of women reported low behavioral risk: 92.7% reported having only one sexual partner, and 91.7% reported no history of transactional sex. This suggests that many women considered to be at low behavioral risk may still be exposed to STIs and HIV—likely through their male partners [28]. This underscores the structural and relational vulnerabilities many women face [29] and highlights the unmet need for targeted HIV testing strategies that include women who may not perceive themselves as at risk but remain vulnerable due to partner behaviors.

While the analysis focused on individual-level risk factors, these findings also reflect deeper structural health determinants. For example, limited access to quality health services in rural areas, unequal distribution of educational resources, and gender dynamics within relationships all influence a woman’s ability to access HIV testing. Addressing these structural barriers is essential to improving testing uptake and achieving broader HIV prevention goals [29].

In this study, marital status was categorized as married, living with a man (consensual union), not in union, divorced/separated, and widowed [20]. In Liberia, formal marriages and cohabiting partnerships are often treated separately because they carry different social and legal implications. While formal marriages are legally recognized and provide women with certain rights, such as inheritance and property ownership, women in consensual unions typically do not have these legal protections. This difference affects various aspects of women’s lives, including their autonomy, financial security, family dynamics, and health decisions, such as whether they get tested for HIV. Research shows that women living with a man face greater challenges when it comes to HIV testing, including less power in household decision-making and more stigma and social barriers compared to those who are married [30].

By combining these two groups in our analysis, we might miss these important differences. When we looked at both formal marriages and consensual unions together, the relationship between marital status and HIV testing appeared weaker. This suggests that lumping these categories together could lead to an underestimation of the unique challenges women in informal unions face when it comes to HIV testing. For more accurate findings, it is crucial to separate these groups and better understand the distinct needs of women in different types of unions.

Education and awareness emerged as significant determinants of HIV testing uptake. Strengthening health literacy through culturally sensitive campaigns, particularly in rural areas, can empower women to seek testing services. School-based HIV education and community outreach, especially among young women and those considered “low-risk” (e.g., monogamous or married), could help normalize testing and reduce stigma. Promoting knowledge of PMTCT and integrating HIV education into maternal health services are also crucial for increasing voluntary testing. Expanding access to testing services is essential. The lower odds of testing among rural women underscore the importance of mobile clinics, community-based testing, and routine integration of HIV testing into reproductive health and antenatal care services. Improving confidentiality, particularly in rural settings, and ensuring respectful, nonjudgmental healthcare experiences can also enhance trust and uptake. These targeted strategies can address structural health barriers and ensure more equitable access to HIV testing for all women, regardless of education level, residence, or perceived risk.

### Strengths and Limitations

Since this study is a secondary data analysis of the DHS, it is representative of the entire population of reproductive-age women in Liberia and inferences made through this study may be generalized to the entire population of Liberian reproductive-aged women. Furthermore, our study had large sample sizes with high response rates. Sample weights were used for this analysis.

However, since this study is a secondary analysis of a cross-sectional study, it automatically restricts us from investigating further on the causal relationships between HIV testing and its risk factors such as religious affiliation in the LDHS 2019–2020, preventing analysis of its potential influence on HIV testing. Also, this study may be exposed to reporting and recall biases as most questions required retrospective data. This might have led to HIV testing outcomes being under-reported or over-reported.

## 5. Conclusions and Recommendations

This study found that the prevalence of HIV testing among Liberian women aged 15 to 49 years in 2020 was 57.17% (95% CI: 56.2 to 60.4). The findings also show that HIV testing among these women was significantly associated with higher educational levels, place of residence, pregnancy history, knowledge of MTCT, and STI history. These factors should be integrated into peer education programs on HIV testing to enhance their effectiveness. However, the relatively low prevalence indicates an unmet need for HIV testing, particularly among women in rural areas, those with limited health services, and less educated women. Therefore, it is recommended that HIV testing and counseling services primarily target these populations. Additionally, future qualitative research focusing on the risk factors for HIV testing would be valuable for further improving HIV testing prevalence.

## Figures and Tables

**Table 1:** Characteristics of study participants among women aged 15–49 years in Liberia.

Variable	Category	Number of Participants (*n*)	Percent of Participants. (%)
Age of a woman	15–19	1250	15.2
	20–24	1387	19.6
	25–29	1200	18.3
	30–34	1051	14.8
	35–39	1103	13.6
	40–44	857	10.2
	45–49	727	8.3
Highest educational level	No Education	2938	32.2
	Primary	2104	22.0
	Secondary	2296	39.8
	Higher education	237	6.0
Place of residence	Urban	4489	61.7
	Rural	3086	38.3
Marital status	Married	2339	28.6
	Not in union	2315	27.5
	Living with a man	2131	34.3
	Widowed	145	1.8
	Divorced/separated	645	7.8
Media exposure	Not at all	6555	78.2
	Less than once a week	228	4.3
	More than once a week	792	17.5
Partner’s highest educational level	No Education	1306	27.8
	Primary	727	12.8
	Secondary	1936	46.9
	Higher education	364	12.5
Employment status	Not employed	5293	67.1
	Employed	2282	32.9
Pregnancy History	No	1167	16.8
	Yes	6408	83.2
Number of sexual partners	1 partner	7083	92.7
	2 partners	470	6.8
	3 or more partners	22	0.6
Transactional Sex	No	1428	91.7
	Yes	115	8.3
STI History	No	5303	66.8
	Yes	2262	33.2
Gender-based violence	No	1675	98.0
	Yes	34	2.0
Discriminatory behavior	No	24	1.2
	Yes	2018	98.8

**Table 2:** Factors associated with HIV testing among women aged 15–49 years in Liberia.

		Bivariate Logistic Regression			Multivariate Logistic Regression		
Variable	Category	OR	95%CI	*p* Value	aOR	95%CI	*p* Value
Age in years	15–19	Ref			Ref		
	20–24	2.53	2.00–3.19	**<0.001**	1.58	1.05–2.38	**0.028**
	25–29	4.33	3.29–5.69	**<0.001**	1.74	1.09–2.79	**0.022**
	30–34	4.27	3.01–6.05	**<0.001**	1.80	1.10–2.96	**0.021**
	35–39	3.80	2.89–5.01	**<0.001**	1.95	1.20–3.17	**0.007**
	40–44	2.88	2.14–3.88	**<0.001**	1.35	0.86–2.12	0.193
	45–49	1.39	1.04–1.87	**0.028**	0.83	0.49–1.40	0.483
Highest educational level	No Education	Ref			Ref		
	Primary	1.67	0.98–1.38	0.076	1.39	1.16–1.68	**<0.001**
	Secondary	1.46	1.24–1.73	**<0.001**	2.10	1.73–2.53	**<0.001**
	Higher education	3.96	2.37–6.65	**<0.001**	6.80	3.75–12.32	**<0.001**
Place of residence	Urban	Ref					
	Rural	0.73	0.62–0.86	**<0.001**	0.82	0.67–0.99	**0.039**
Marital status	Married	Ref			Ref		
	Not in union	0.70	0.60–0.83	0.110			
	Living with a man	1.31	1.09–1.58	**0.005**	1.35	1.03–1.51	**<0.001**
	Widowed	0.73	0.47–1.12	0.148			
	Divorced/separated	1.47	1.14–1.90	**0.004**	1.37	1.05–1.79	**0.019**
Media exposure	Not at all	Ref			Ref		
	Less than once a week	1.54	1.03–2.32	**0.037**	0.81	0.36–1.81	0.606
	More than once a week	1.94	1.56–2.43	**<0.001**	1.66	0.91–3.00	0.096
Partner’s highest educational level	No Education	Ref			Ref		
	Primary	1.28	1.02–1.63	**0.037**	1.19	0.91–1.55	0.210
	Secondary	1.79	1.47–2.18	**<0.001**	1.41	1.13–1.75	**0.002**
	Higher education	3.24	2.24–4.71	**<0.001**	1.82	1.13–2.93	**0.013**
Employment status	Not employed	Ref			Ref		
	Employed	1.25	1.09–1.45	**0.002**	1.02	0.81–1.30	0.814
Pregnancy History	No	Ref					Ref
	Yes	4.75	3.89–5.83	**<0.001**	6.40	4.99–8.22	**<0.001**
Number of sexual partners	1 partner	Ref					
	2 partners	1.05	0.84–1.32	0.685			
	3 or more partners	0.40	0.13–1.21	0.105			
Transactional Sex	No	Ref					
	Yes	1.28	0.75–2.19	0.362			
STI History	No	Ref					Ref
	Yes	1.24	1.07–1.45	**0.006**	1.21	1.02–1.50	**0.030**
Knowledge of drugs to avoid MTCT	No	Ref					
	Yes	1.72	1.35–2.20	***p* < 0.001**	1.65	1.23–2.21	**0.001**
Gender-based violence	No	Ref					
	Yes	0.93	0.36–2.37	0.880			
Discriminatory behavior	No	Ref					
	Yes	1.49	0.44–5.04	0.515			

OR = odds ratio, aOR = adjusted odds ratio, CI = confidence interval, *p* value threshold = 0.01, bold lettered *p* value represents the statistically significant values

## Data Availability

The DHS data is publicly available at www.dhsprogram.com, accessed 20 April 2023.
